# Time-resolved hypothalamic open flow micro-perfusion reveals normal leptin transport across the blood–brain barrier in leptin resistant mice

**DOI:** 10.1016/j.molmet.2018.04.008

**Published:** 2018-04-27

**Authors:** Maximilian Kleinert, Petra Kotzbeck, Thomas Altendorfer-Kroath, Thomas Birngruber, Matthias H. Tschöp, Christoffer Clemmensen

**Affiliations:** 1 Institute for Diabetes and Obesity, Helmholtz Diabetes Center, Helmholtz Zentrum München, German Research Center for Environmental Health (GmbH), Neuherberg, Germany; 2 Division of Metabolic Diseases, Department of Medicine, Technische Universität, Munich, Germany; 3 Section for Molecular Physiology, Department of Nutrition, Exercise and Sports, Faculty of Science, University of Copenhagen, Copenhagen, Denmark; 4 Division of Endocrinology and Diabetology, Medical University of Graz, Graz, Austria; 5 JOANNEUM Research GmbH, HEALTH – Institute for Biomedicine and Health Sciences, Neue Stiftingtalstrasse 2, 8010 Graz, Austria; 6 Novo Nordisk Foundation Center for Basic Metabolic Research, Faculty of Health and Medical Sciences, University of Copenhagen, Copenhagen, Denmark

**Keywords:** Obesity, Hypothalamus, Leptin, Leptin resistance, Blood–brain barrier, Leptin transport

## Abstract

**Objective:**

The inability of leptin to suppress food intake in diet-induced obesity, sometimes referred to as leptin resistance, is associated with several distinct pathological hallmarks. One prevailing theory is that impaired transport of leptin across the blood–brain barrier (BBB) represents a molecular mechanism that triggers this phenomenon. Recent evidence, however, has challenged this notion, suggesting that leptin BBB transport is acquired during leptin resistance.

**Methods:**

To resolve this debate, we utilized a novel cerebral Open Flow Microperfusion (cOFM) method to examine leptin BBB transport in male C57BL/6J mice, fed a chow diet or high fat diet (HFD) for 20 days.

**Results:**

Basal plasma leptin levels were 3.8-fold higher in HFD-fed mice (p < 0.05). Leptin administration (2.5 mg/kg) elicited similar pharmacokinetic profiles of circulating leptin. However, while leptin reduced food intake by 20% over 22 h in chow-fed mice, it did not affect food intake in HFD-fed mice. In spite of this striking functional difference, hypothalamic leptin levels, as measured by cOFM, did not differ between chow-fed mice and HFD-fed mice following leptin administration.

**Conclusions:**

These data suggest that leptin transport across the BBB is not impaired in non-obese leptin resistant mice and thus unlikely to play a direct role in the progression of pharmacological leptin resistance.

## Highlights

• Cerebral Open Flow Microperfusion can be utilized to assess hypothalamic leptin levels.
• Twenty days of high-fat diet feeding causes functional resistance to exogenous leptin.
• Transport of leptin across the BBB is not impaired by HFD-induced leptin resistance.

## 1 Introduction

Leptin is a 16 kDa hormone that is secreted from white adipocytes to signal satiety in the brain. It plays a critical role in the neuroendocrine regulation of body-weight [1], [2], [3]. Null-mutations in the leptin-encoding gene cause morbid obesity in mice and humans [3], [4]. This monogenetic obesity is corrected by treatment with recombinant leptin [3], [5]. In contrast, common polygenetic obesity [6] is associated with increased leptin levels and treatment with additional leptin has limited efficacy to lower body-weight under these circumstances [7], [8], [9]. One prevailing theory is that impaired transport of leptin across the blood–brain barrier (BBB) plays a central role to this blunted responsiveness to exogenous leptin – sometimes referred to as *leptin resistance.* This theory of a defect in leptin transport across the BBB is rooted in several observations. First, early studies suggested a lower ratio of leptin in the cerebral spinal fluid (CSF) to serum leptin in obese humans [10], [11], possibly reflecting saturation in leptin transport. Second, rodents fed a high-fat diet develop resistance to peripherally administered leptin before they develop resistance to centrally injected leptin [12], [13]. Third, using radiolabeled leptin, researchers found a reduced brain-to-serum ratio of leptin in obese relative to lean animals [14].

However, leptin resistance describes a complex phenomenon that can emerge from several distinct entry points. Beyond impairments in leptin transport, perturbations in the leptin receptor signaling cascade and disruptions in neural circuits downstream of leptin target neurons can result in leptin resistance [9], [15]. The relative contribution and the sequential manifestation of these events reported to be associated with functional leptin resistance remain unclear. Given this uncertainty, we took advantage of recent methodological advances to specifically quantify leptin transport across the BBB in the early stages of diet-induced leptin resistance (i.e. the inability of exogenous leptin to suppress food intake). Cerebral Open Flow Microperfusion (cOFM) is a new *in vivo* technique that allows for continuous sampling of the interstitial fluid in brain tissue [16], [17], [18], enabling the time-resolved assessment of leptin BBB transport in response to peripheral administration of recombinant leptin. Using simultaneous measurements of plasma leptin levels, we generated the first combined profile of peripheral and hypothalamic leptin pharmacokinetics examining diet-induced leptin resistance *in vivo*.

## 2 Materials and methods

### 2.1 Animals

All animal experiments were approved either by the Danish Animal Experimental Inspectorate or the Austrian Federal Government (BMWFW-66.010/0035-WF/V/3b/2017) and were performed in accordance with Directive 2010/63/EU on the protection of animals used for scientific purposes. Mice were housed on a 12:12-h light-dark cycle at 21–22 °C.

### 2.2 Assessment of pharmacological leptin responsiveness

Fourteen-week-old male C57BL6/J mice were maintained on a standard rodent chow diet and were single-housed 14 days before a diet switch, for which mice were randomized to remain on chow diet or to receive high-fat diet (Research diets, D12331) for 20 days. Mice had *ad libitum* access to food and water. At 5 pm on day 20, mice were injected intraperitoneally with either a bolus of recombinant mouse leptin (2.5 mg/kg; R&D Systems, USA) or a vehicle solution. Food was weighed at 2, 6, 14, and 22 h after the injection and the difference to pre-injection food was defined as food intake.

### 2.3 Surgery and implantation

Body-weight matched 13 -16-week-old male C57BL6/J mice were anesthetized with fentanyl (50 μg/kg, Hameln Pharma, Germany), midazolam (5 mg/kg, Erwo Pharma, Austria) and medetomidin (5 mg/kg, Orion Pharma, Austria). During stereotactic surgeries and probe implantation, anesthesia was maintained by constant inhalation of oxygen (1.5 L/min) and isoflurane (0.5% - 1%, Forane®, Abbott, Germany). The cOFM probe was implanted into the hypothalamus using the following coordinates: AP 0.7 mm, ML -1.5 mm, DV -6 mm from bregma. For post-surgical pain management, animals received an antibiotic (Baytril, Bayer, Germany) and rimadyl (Carprofen; Zoetis, Austria). After surgery, animals recovered for 14 days; then animals were either kept on chow diet or switched to high-fat diet (Research diets, D12331) for 20 days.

### 2.4 Interstitial fluid sampling

Animals were anaesthetized by isoflurane (0.5%, 1.5 L/h O_2_) and placed on a homeothermic blanket. Body temperature was maintained at 38 °C. The healing dummy was replaced by the sampling inlet, which was connected to syringe pumps. The cOFM probe was perfused with artificial and sterile cerebrospinal fluid (aCSF) in push–pull mode. Prior to sampling, the system was equilibrated by a flush sequence using a flow rate of 2 μl/min for 5 min and a run-in sequence with 0.5 μl/min for 30 min. During the sampling procedure flow rate was constant at 0.5 μl/min. After baseline sampling of 30 min, mice received a bolus of 2.5 mg/kg recombinant mouse leptin (R&D Systems, USA) intraperitoneally. Thereafter, cOFM samples were collected over 6 h and stored at −80 °C. Corresponding blood samples were withdrawn through the tail vein into lithium-heparin coated tubes and centrifuged for 5 min at 2000 g. Plasma was stored at −80 °C. After the last sampling time point, mice were perfused with 10% neutrally buffered formalin, and brains were carefully dissected out and post-fixed overnight. Afterward, brains were kept in 1× PBS. Prior to sectioning, brains were bathed in 30% sucrose for at least 24 h. 30 μm thick sections from the entire hypothalamus were made and stained using a standard hematoxylin and eosin (H&E) protocol to verify proper probe placement.

### 2.5 Leptin analysis

Leptin was analyzed with a Leptin ELISA kit (Cat # 22-LEPMS-E01 from ALPCO, Salem, USA) according to the manufacturer's instructions.

### 2.6 Statistical analysis

Data are reported as means ± SEM. Graphs were prepared in Prism version 7.0 (GraphPad Software, San Diego, CA, USA). Data were analyzed with SigmaPlot version 13.0 (SYSTAT Software, San Jose, CA, USA). The type of statistical analysis performed is stated in the figure legends. p < 0.05 was considered statistically significant.

## 3 Results

### 3.1 Twenty days of HFD-feeding induces functional leptin resistance

**Figure 1:**
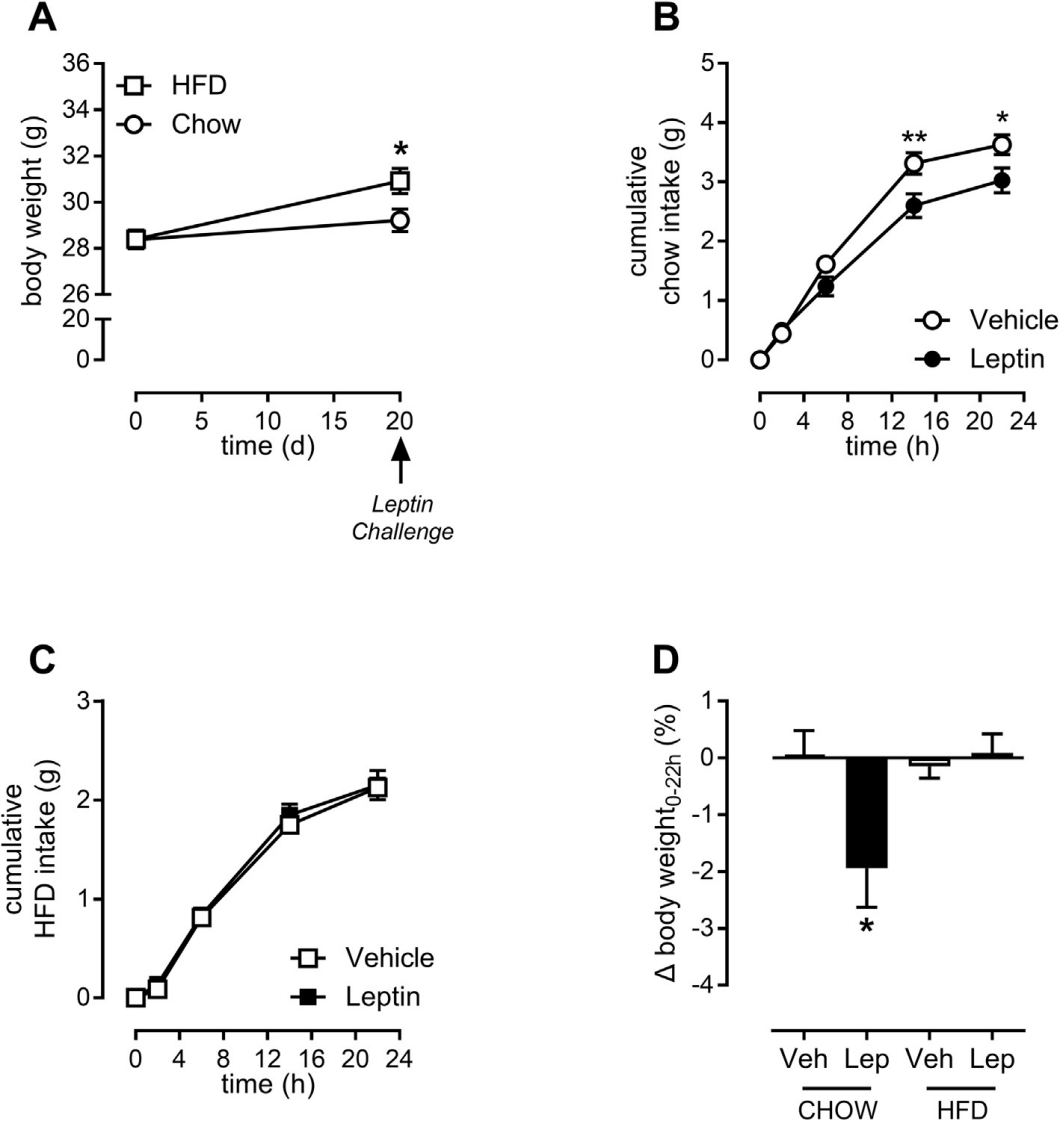
**Twenty days of HFD-feeding induces functional leptin resistance.** Mice, fed either chow or HFD for 20 days, were intraperitoneally injected with recombinant leptin or vehicle at 5 pm on day 20. Body-weights on day 0 and on day 20 before the injections were recorded (A). Food weight was determined at indicated times, and cumulative food intake was calculated (B, C). Body-weights were measured 22 h after the injections and delta (Δ) body-weight was calculated (D). Data are means ± SEM and n = 7–8. Data in panels A, B and C were analyzed by two-way repeated measures analysis of variance and a Holm-Sidak post-hoc test. In panel D, the differences between vehicle (Veh) and leptin (Lep) within each diet were analyzed by a two-tailed Student's t-test. *p < 0.05, **p < 0.01 between vehicle and leptin.

Compared to chow-fed mice, HFD-fed mice did gain 1.7 g more body-weight (p < 0.05) but did not reach an obese state by any definition (Figure 1A). At 5 pm on day 20, mice were injected with recombinant leptin (2.5 mg/kg) or vehicle solution. In chow-fed mice, leptin decreased food intake, measured over 22 h, by ∼20% (p < 0.05) compared to vehicle-injected mice (Figure 1B). Leptin decreased body-weight by 2% compared to the vehicle-injected mice (p < 0.05) (Figure 1D). In contrast to its effect in chow-fed animals, leptin injection in HFD-fed mice had no effect on food intake or body-weight (Figure 1C). These results demonstrate that 20 days of HFD feeding induces functional leptin resistance.

### 3.2 Hypothalamic availability of exogenous leptin is unaltered by leptin resistance

**Figure 2:**
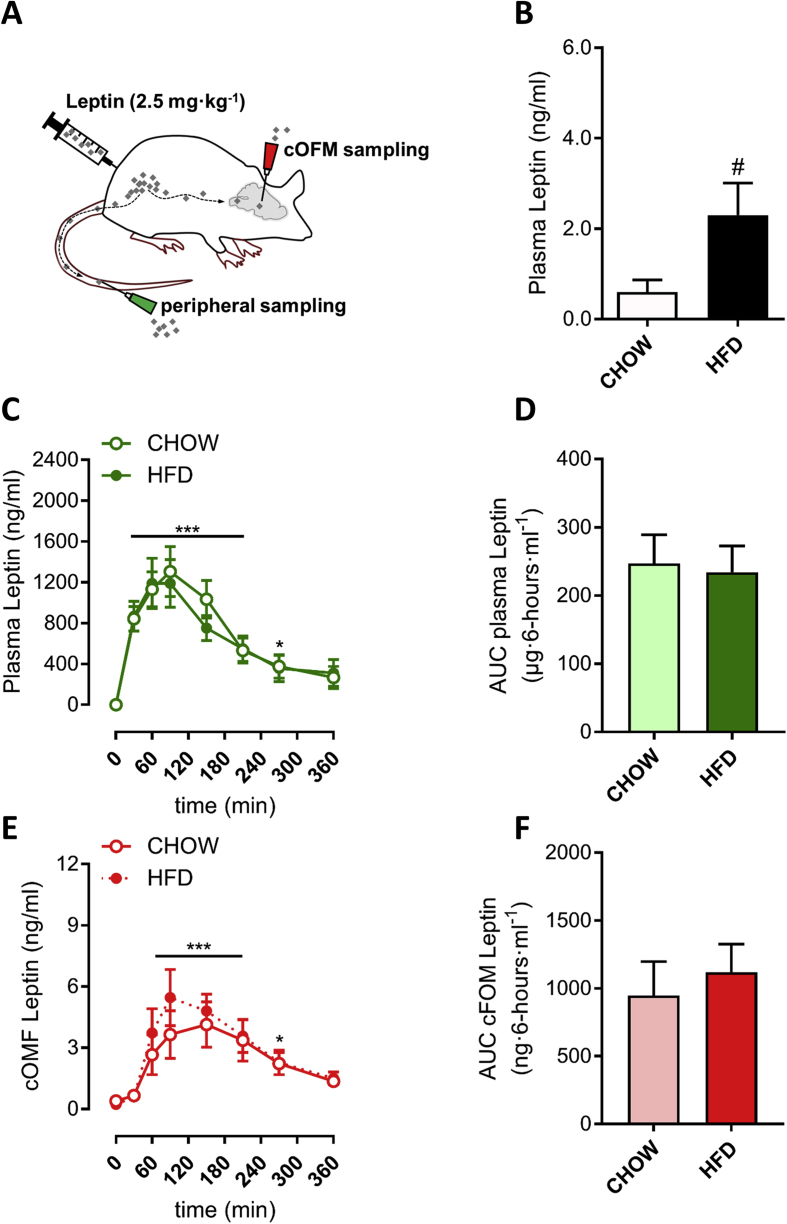
**The rate of leptin appearance in the brain is normal in leptin resistant mice.** Mice with implanted cOFM probes (see methods) were fed either chow or HFD for 20 days before leptin transport into the brain was assessed (A). Prior to the intraperitoneal injection of recombinant leptin, mixed tail blood was collected to determine baseline plasma leptin levels (B). Leptin concentrations were then determined in either plasma (C) or interstitial fluid collected from the brain (E) at indicated time-points. Corresponding area under the curves (AUCs) were calculated (D,F). Data are means ± SEM and n = 10 for all groups. Data in panel B, D and E were analyzed with a two-tailed Student's t-test; data in panel C and E were analyzed by two-way repeated measures analysis of variance and a Holm-Sidak post-hoc test was performed for the variable ‘time’. *p < 0.05, ***p < 0.001 compared to 0 min ^#^p < 0.05 compared to chow.

**Figure S1:**
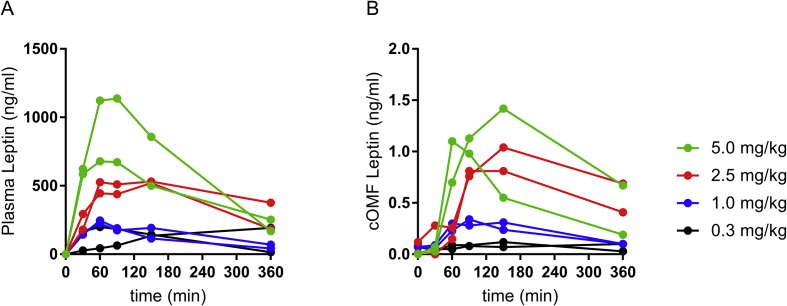
Circulating (A) and cOFM (B) leptin concentration prior to (0 min) and at indicated time points after intraperitoneal injection of indicated doses of leptin.

**Figure S2:**
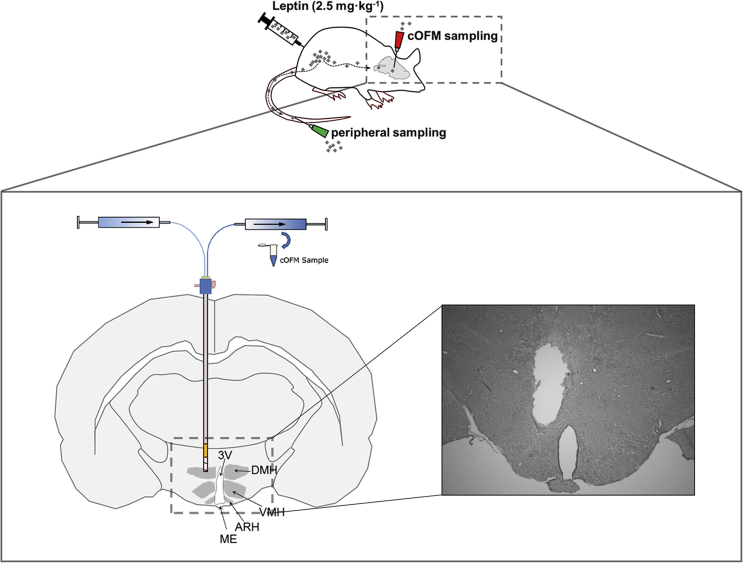
Schematic of the cOFM probe placement along with coronal H&E stained image of the hypothalamus.

Before assessing leptin BBB transport (as described in the methods; also see schematics in Figure 2A and Suppl. Figure 2), baseline plasma levels of endogenous leptin were 3.8-fold higher in HFD-fed mice (p < 0.05; Figure 2B). Following peripheral injection of recombinant leptin (2.5 mg/kg), circulating leptin levels increased in a similar manner in chow-fed and HFD-fed mice, reaching a peak of ∼1200 ng/ml at 60 min after the injection (Figure 2C) (2.5 mg/kg leptin dose, was based on a dose-titration study (Suppl. Figure 1A–B). The pharmacokinetic profiles of leptin were similar between chow- and HFD-fed mice, which is also reflected by AUCs of 247 ± 42 and 234 ± 39 μg·6-hours·ml^−1^. These data indicate that during the 6-hour period after injection, the peripheral leptin bioavailability was comparable in chow- and HFD-fed mice. In parallel, we analyzed leptin levels over time in interstitial fluid obtained from the hypothalamus by cOFM sampling (Figure 2A). The rate of leptin appearance in the hypothalamus was similar between chow- and HFD-fed mice, with peak concentrations of 4.0–5.5 ng⋅ml^−1^ at 60–120 min post-injection. Similar to the peripheral leptin levels, the overall pharmacokinetic profile of hypothalamic leptin showed no difference between the two diet groups with AUCs of 949 ± 249 and 1121 ± 204 ng·6-hours·ml^−1^ for chow- and HFD-fed mice, respectively (p = 0.60, Figure 2F). Collectively, these data indicate that leptin transport across the BBB remains normal in HFD-fed mice with reduced leptin responsiveness, eliminating that mechanism as a potential cause for leptin resistance.

## 4 Discussion

By using a methodological innovation (cOFM) that allows for time-resolved *in vivo* sampling of hypothalamic interstitial fluid, we revealed, for the first time, the pharmacokinetic and pharmacodynamic profiles of exogenous leptin in the mouse brain and demonstrated that transport of leptin across the BBB is not impaired in HFD-induced leptin resistance.

Since the discovery of leptin in 1994 and the delineation of its anorectic effects, there has been a considerable interest in transforming these insights into efficacious anti-obesity pharmacotherapy. However, most obese individuals are hyperleptinemic and leptin therapy has limited weight-lowering effects [7]. Recent evidence, however, concludes that a series of distinct pharmacological compounds can potentiate leptin signaling and empower its weight-lowering abilities in HFD-induced obesity [19]. Before such leptin-based combination therapies are tested in clinical studies, more basic research on leptin physiology and leptin pharmacology is required.

It is unclear, for instance, whether diminished leptin transport into the brain is causal or secondary in the development of leptin resistance. Therefore, we assessed leptin transport in the early phase of HFD-diet induced leptin resistance, when patent obesity is absent. The use of cOFM allowed for repeated sampling of hypothalamic interstitial fluids from the same mouse enabling us to assess central leptin appearance/availability over time. This is an improvement over previous studies, in which mice are sacrificed at each desired time-point to determine abundance of labeled leptin (e.g. radioactive or fluorescent leptin) in the brain. Thus, we demonstrate that leptin transport across the BBB is normal in the face of functional leptin resistance after 20 days of HFD.

In contrast to these data, a prevailing model proposes that impaired leptin transport across the BBB triggers the development of leptin resistance in obesity [20]. Thus, when it was found that the ratio of leptin in the CSF to leptin in circulation is lower with obesity [10], [11] it was proposed that this reflects a saturation/impairment in leptin transport across the BBB that drives leptin resistance. Yet, the same studies showed that total levels of leptin in the CSF are higher with obesity, indicating the existence of a central component in leptin resistance. It is an equally plausible explanation that leptin resistance and resulting central hyperleptinemia trigger adaptations to actually decrease leptin transport rates across the BBB to lower the abundance of leptin in the brain. Chronic central hyperleptinemia can cause maladaptations, like hypertension due to leptin's action on leptin receptors (LEPRs) in the dorsomedial hypothalamic nucleus [21].

Therefore, our findings do not preclude that defects in leptin transport are acquired with chronic obesity (i.e. with chronic leptin resistance). After 4 weeks of HFD feeding, peripheral leptin injection induced hypothalamic phosphorylation of STAT3 (p-STAT3) [22], a key mediator of the central action of leptin [23]. In contrast, after 15 weeks of HFD feeding induction of p-STAT3 by peripheral leptin was impaired [22]. At the same time, intracerebroventricular (ICV) infusion of leptin demonstrated that the capacity for p-STAT3 induction is intact following 15 weeks of HFD-feeding [22], indicating that a defect in leptin transport is present during the later stages of leptin resistance. In addition, rats selectively bred to develop diet-induced obesity were functionally leptin resistant at age 4 weeks, despite normal leptin transport across the BBB [24]. At age 23 weeks, however, leptin BBB transport was reduced in these rats [24]. Similarly, the first study to demonstrate defects in leptin BBB transport with obesity was conducted in one-year old outbred CD1 mice selected for leaness and heaviness [14]. Taken together, our data and the above reports suggest that defects in leptin BBB transport are acquired rather than causal, indicating that central leptin signaling defects are at the root of the etiology of leptin resistance.

This is supported by animal studies consistently showing that leptin resistance coincides with defects in signaling transmission distal to LEPR, even when leptin is administered ICV [25]. The observation that hyperleptinemia *per se*, and thus chronically higher LEPR signaling, is upstream of leptin resistance in obesity could imply a pharmacological ceiling effect. This is in agreement with elevated hypothalamic pSTAT3 in obesity [26] and the fact that LEPR antagonism increases food intake and body-weight equally in lean and DIO mice [27] – despite differences in exogenous leptin sensitivity. Thus, while hypoleptinemia serves as a hunger-promoting signal, it remains enigmatic if increasing leptin in the context of a positive energy balance adds relevant catabolic signals [25], [28].

Our study has certain limitations. The cOFM method is an invasive procedure and the probe diameter of 500 μm is relatively large compared to structures of the mouse brain. Therefore, altered function due to trauma in the implanted brain area cannot be excluded, which is why complementary future studies, using less invasive methods, are warranted to consolidate our conclusions. At the same time, a major advantage of the cOFM method is that it allows for continuous *in vivo* sampling of interstitial fluids in different compartments of the brain (cortex, striatum, hypothalamus). In previous studies, we have also demonstrated that the cOFM probe causes minimal tissue trauma compared to other sampling techniques (e.g., microdialysis) [16], [18], [29], [30] and given the large pore size of the exchange area of the cOFM probe, the sampled analytes are not limited to a specific size or chemical property. It must also be noted that our study focused on leptin actions in response to exogenous leptin administration. Thus, we cannot draw any conclusions about the transport dynamics of endogenous leptin across the BBB.

In summary, we have created the first time-resolved pharmacokinetic- and pharmacodynamic profile of exogenous leptin in the hypothalamus, comparing leptin sensitive and leptin resistant mice. Our data demonstrate that leptin transport into the hypothalamus is normal in HFD-induced leptin resistance, thereby removing impaired BBB transport as a direct cause for diet-induced reductions in leptin responsiveness.

## Author contributions

M.K. co-conceptualized the project, researched and interpreted data, co-wrote, reviewed and edited the manuscript. P.K., T. A-K., T.B., researched data and reviewed the manuscript. M.H.T co-conceptualized the project and co-wrote the manuscript. C.C. co-conceptualized the project, researched and interpreted data, co-wrote, reviewed and edited the manuscript. C.C. is the guarantor of this work and, as such, had full access to all the data in the study and takes responsibility for the integrity of the data and the accuracy of the analyses.

## References

[1] Zhang Y., Proenca R., Maffei M., Barone M., Leopold L., Friedman J.M. (1994). Positional cloning of the mouse obese gene and its human homologue. Nature.

[2] Maffei M., Halaas J., Ravussin E., Pratley R.E., Lee G.H., Zhang Y. (1995). Leptin levels in human and rodent: measurement of plasma leptin and ob RNA in obese and weight-reduced subjects. Nature Medicine.

[3] Campfield L.A., Smith F.J., Guisez Y., Devos R., Burn P. (1995). Recombinant mouse OB protein: evidence for a peripheral signal linking adiposity and central neural networks. Science.

[4] Montague C.T., Farooqi I.S., Whitehead J.P., Soos M.A., Rau H., Wareham N.J. (1997). Congenital leptin deficiency is associated with severe early-onset obesity in humans. Nature.

[5] Farooqi I.S., Jebb S.A., Langmack G., Lawrence E., Cheetham C.H., Prentice A.M. (1999). Effects of recombinant leptin therapy in a child with congenital leptin deficiency. New England Journal of Medicine.

[6] Considine R.V., Sinha M.K., Heiman M.L., Kriauciunas A., Stephens T.W., Nyce M.R. (1996). Serum immunoreactive-leptin concentrations in normal-weight and obese humans. New England Journal of Medicine.

[7] Heymsfield S.B., Greenberg A.S., Fujioka K. (1999). Recombinant leptin for weight loss in obese and lean adults: a randomized, controlled, dose-escalation trial. Journal of the American Medical Association.

[8] Frederich R.C., Hamann A., Anderson S., Löllmann B., Lowell B.B., Flier J.S. (1995). Leptin levels reflect body lipid content in mice: evidence for diet-induced resistance to leptin action. Nature Medicine.

[9] Myers M.G., Heymsfield S.B., Haft C., Kahn B.B., Laughlin M., Leibel R.L. (2012). Defining clinical leptin resistance - challenges and opportunities. Cell Metabolism.

[10] Caro J.F., Kolaczynski J.W., Nyce M.R., Ohannesian J.P., Opentanova I., Goldman W.H. (1996). Decreased cerebrospinal-fluid/serum leptin ratio in obesity: a possible mechanism for leptin resistance. The Lancet.

[11] Schwartz M.W., Peskind E., Raskind M., Boyko E.J., Porte J. (1996). Cerebrospinal fluid leptin levels: relationship to plasma levels and to adiposity in humans. Nature Medicine.

[12] Van Heek M., Compton D.S., France C.F., Tedesco R.P., Fawzi A.B., Graziano M.P. (1997). Diet-induced obese mice develop peripheral, but not central, resistance to leptin. Journal of Clinical Investigation.

[13] Halaas J.L., Boozer C., Blair-West J., Fidahusein N., Denton D.A., Friedman J.M. (1997). Physiological response to long-term peripheral and central leptin infusion in lean and obese mice. Proceedings of the National Academy of Sciences of the U S A.

[14] Banks W.A., DiPalma C.R., Farrell C.L. (1999). Impaired transport of leptin across the blood-brain barrier in obesity. Peptides.

[15] Friedman J. (2016). The long road to leptin. Journal of Clinical Investigation.

[16] Birngruber T., Ghosh A., Perez-Yarza V., Kroath T., Ratzer M., Pieber T.R. (2013). Cerebral open flow microperfusion: a new in vivo technique for continuous measurement of substance transport across the intact blood-brain barrier. Clinical and Experimental Pharmacology and Physiology.

[17] Birngruber T., Sinner F. (2016). Cerebral open flow microperfusion (cOFM) an innovative interface to brain tissue. Drug Discovery Today: Technologies.

[18] Ghosh A., Birngruber T., Sattler W., Kroath T., Ratzer M., Sinner F. (2014). Assessment of blood-brain barrier function and the neuroinflammatory response in the rat brain by using cerebral open flow microperfusion (cOFM). PLoS One.

[19] Quarta C., Sánchez-Garrido M.A., Tschöp M.H., Clemmensen C. (2016). Renaissance of leptin for obesity therapy. Diabetologia.

[20] Banks W.A. (2016). From blood−brain barrier to blood−brain interface: new opportunities for CNS drug delivery. Nature Reviews Drug Discovery.

[21] Simonds S.-E., Pryor J., Ravussin E., Greenway F.-L., Dileone R., Allen A.-M. (2014). Leptin mediates the increase in blood pressure associated with obesity. Cell.

[22] El-Haschimi K., Pierroz D.D., Hileman S.M., Bjørbæk C., Flier J.S. (2000). Two defects contribute to hypothalamic leptin resistance in mice with diet-induced obesity. Journal of Clinical Investigation.

[23] Bates S.H., Stearns W.H., Dundon T.A., Schubert M., Tso A.W.K., Wang Y. (2003). STAT3 signalling is required for leptin regulation of energy balance but not reproduction. Nature.

[24] Levin B.E., Dunn-Meynell A.A., Banks W.A. (2004). Obesity-prone rats have normal blood-brain barrier transport but defective central leptin signaling before obesity onset. American Journal of Physiology - regulatory, Integrative and Comparative Physiology.

[25] Pan W.W., Myers M.G. (2018). Leptin and the maintenance of elevated body weight. Nature Reviews Neuroscience.

[26] Münzberg H., Flier J.S., Bjørbæk C. (2004). Region-specific leptin resistance within the hypothalamus of diet-induced obese mice. Endocrinology.

[27] Ottaway N., Mahbod P., Rivero B., Norman L.A., Gertler A., D'Alessio D. (2015). Diet-induced obese mice retain endogenous leptin action. Cell Metabolism.

[28] Flier J.S., Maratos-Flier E. (2017). Leptin's physiologic role: does the emperor of energy balance have No clothes?. Cell Metabolism.

[29] Birngruber T., Ghosh A., Hochmeister S., Asslaber M., Kroath T., Pieber T.R. (2014). Long-Term implanted cOFM probe causes minimal tissue reaction in the brain. PLoS One.

[30] Birngruber T., Raml R., Gladdines W., Gatschelhofer C., Gander E., Ghosh A. (2014). Enhanced doxorubicin delivery to the brain administered through glutathione PEGylated liposomal doxorubicin (2B3-101) as compared with generic caelyx/doxil - a cerebral open flow microperfusion pilot study. Journal of Pharmaceutical Sciences.

